# Perioperative and oncologic outcomes of robotic surgery for pediatric solid abdominal tumors: a single-center 10-year experience

**DOI:** 10.3389/fped.2025.1453718

**Published:** 2025-03-13

**Authors:** Ashitosh Pokharkar, Priyank Yadav, Deepak K. Kandpal, Amita Mahajan, Sujit Kumar Chowdhary

**Affiliations:** ^1^ Indraprastha Apollo Hospitals, New Delhi, India; ^2^ Department of Urology and Renal Transplantation, Sanjay Gandhi Post Graduate Institute of Medical Sciences (SGPGI), Lucknow, Uttar Pradesh, India

**Keywords:** pediatric cancer, radical nephrectomy, minimally invasive surgery, robotic surgery, adrenalectomy

## Abstract

**Introduction:**

Minimally invasive surgery (MIS) has revolutionized oncological surgery with benefits such as smaller incisions and quicker recovery. However, its use in pediatric population is debated due to concerns about complete tumor resection and complications. Robotic surgery, offering enhanced visualization and precision, may address these issues. This study reviews a decade of robotic surgery for pediatric solid abdominal tumors at a single center, assessing perioperative and oncological outcomes.

**Methods:**

This prospective, single-arm study involved patients aged over six months, treated between 2013 and 2023 for solid abdominal tumors. Exclusion criteria included weight <6 kg, distant metastasis, and tumors >6 cm in shortest diameter. All patients underwent thorough preoperative assessment, including imaging and multidisciplinary evaluation. Surgeries were performed using the da Vinci Si Surgical System and data on patient demographics, perioperative outcomes, and follow-up were systematically collected.

**Results:**

The study cohort included 20 patients (9 boys and 11 girls) with a median age of 3.5 years. The median operative time was 114 min, with a median hospital stay of 3 days. Conversion to open surgery was necessary in 10% of cases. R0 resection was achieved in all cases, with a satisfactory lymph node sampling. Median follow-up of 5 years showed overall survival and event-free survival rates of 90%.

**Conclusion:**

Robotic surgery for pediatric abdominal tumors is safe and effective, reducing blood loss and hospital stays without compromising oncological outcomes. Proper case selection and adherence to oncological principles are essential. Further multicenter studies are needed to validate these findings and optimize the use of robotic surgery in pediatric oncology.

## Introduction

The advent of minimally invasive surgical (MIS) techniques has transformed oncological surgery, providing significant benefits compared to conventional open surgical methods. MIS procedures involve making smaller incisions, resulting in less postoperative discomfort for patients which allows for faster recovery times and shortened hospital stays besides improved cosmetic results (1). While MIS techniques have been widely adopted for various procedures in children, their application in pediatric oncological surgery remains a subject of ongoing debate. Some experts argue that the fundamental principles of oncologic surgery, such as adequate exposure, complete resection, and adherence to surgical margins, may limit the suitability of MIS (2, 3). Conversely, others have reported comparable outcomes between MIS and open approaches, suggesting that these concerns can be mitigated with proper surgical technique and experience (4).

Conventional laparoscopic MIS for solid tumor removal in children poses distinct technical challenges, including restricted operating space, limited tumor exposure, difficulties in dissection and tissue manipulation, ensuring protection of vital structures, and the risk of tumor spillage or seeding (2, 5). Robotic surgery has emerged as a promising alternative, offering the potential to overcome these limitations. The robotic platform provides three-dimensional, high-definition stereoscopic visualization, enhanced dexterity through wristed instrumentation, and motion scaling capabilities, facilitating precise dissection and manipulation in confined spaces (6). Furthermore, the accuracy and dexterity afforded by robotic instrumentation make it highly effective in accessing challenging anatomical regions that may be difficult to reach with conventional laparoscopy (7). However, robotic surgery in the pediatric population also faces several challenges. The size of trocars is a significant issue, as standard robotic instruments are often too large for small pediatric patients, leading to difficulties in achieving adequate intra-abdominal working space. Challenging trocar positioning is another concern, particularly in infants and neonates, where the limited anatomical space complicates optimal placement and increases the risk of injury. Additionally, the number of robotic arms used can be problematic; using multiple arms in a confined space can lead to collisions and further restrict maneuverability. These factors collectively limit the widespread adoption and efficacy of robotic surgery in pediatric patients.

While early attempts at evaluating the outcomes of MIS in pediatric tumor surgery were hindered by inadequate patient enrollment (8), a growing body of literature, comprising case reports and series, has demonstrated the feasibility and safety of employing MIS techniques, including robotic surgery, in pediatric oncosurgery (9, 10). This article presents our institution's decade-long experience in managing pediatric solid tumors using robotic surgery. We analyze the role and applicability of this approach, evaluate its benefits and associated complications, and compare our results with published long-term outcomes for pediatric solid tumors.

## Methods

### Study design and patient selection

This prospective, single-arm study aimed to evaluate the role of robotic surgery in managing pediatric solid abdominal tumors over a 10-year period, from 2013 to 2023, at our university hospital. The inclusion criteria were patients aged greater than 6 months, diagnosed with solid abdominal tumors, irrespective of their preoperative or postoperative chemotherapy status, with a shortest tumor diameter of up to 6 cm (to avoid larger incisions for specimen retrieval). Patients were excluded if they met any of the following criteria: (a) weight less than 6 kg, (b) presence of distant metastasis, or (c) tumors with a shortest diameter larger than 6 cm. Based on the preceding 10-year data, with an annual volume of 10–15 cases of abdominal solid tumors and approximately 15%–20% of cases being performed robotically, the target enrollment was 20 patients over the study period.

### Preoperative evaluation and staging

Each patient underwent a comprehensive evaluation by a pediatric oncologist and a multidisciplinary tumor board. The extent of the tumor, both local and distant, was determined using computed tomography (CT) and/or magnetic resonance imaging (MRI). Positron emission tomography-computed tomography (PET-CT) was utilized as deemed necessary by the tumor board for proper assessment of the extent of disease and resectability.

### Disease-specific protocols and management

Patients with Wilms tumor (WT) were treated according to the International Society of Pediatric Oncology's (SIOP) protocols. Image-Defined Risk Factors (IDRFs) were established at diagnosis and after neoadjuvant chemotherapy for neuroblastomas (NBTs). Metaiodobenzylguanidine (MIBG) scans were performed to assess disease extent at diagnosis for NBTs. Patients with pheochromocytoma (PCC) were optimized in the pediatric intensive care unit (PICU) prior to surgery, with appropriate management of blood pressure, fluid status, and any associated complications.

### Surgical approach

**Figure 1:**
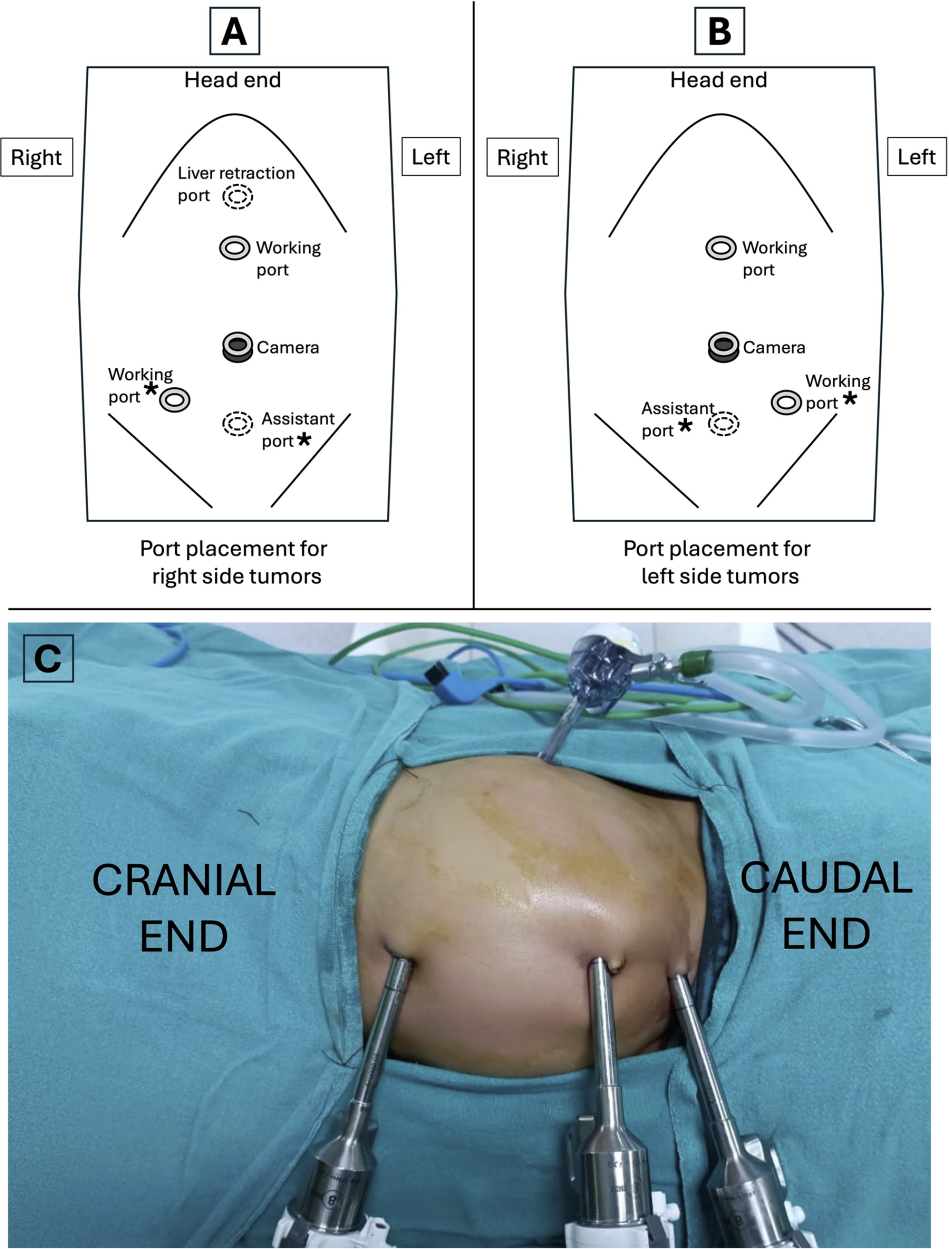
Port placement for robotic-assisted procedures. **(A)** Port placement for right side tumors. **(B)** Port placement for left side tumors. **(C)** Intraoperative image showing port placement for a left sided tumor in a child. Asterisks (*) in **(A** and **B)** indicate the ports that can be used interchangeably across different versions of the surgical robot.

All procedures were performed using a transperitoneal approach with 8 mm ports for the camera and working instruments. A 5 mm assistant port was added between the umbilicus and the pubic symphysis (Figure 1). Patients undergoing nephrectomy or adrenalectomy were positioned in a lateral decubitus position, with one robotic port midway between the umbilicus and the xiphoid and the other in the iliac fossa ipsilateral to the affected organ. For other procedures, patients were placed in the supine position with standard port placement using the triangulation principle. A fifth 5 mm port was utilized for liver retraction during right adrenalectomy. Intraoperative ultrasound and frozen section capabilities were available for all cases.

### Surgical technique and hemostasis

The steps of nephrectomy or adrenalectomy were identical to the standard robotic transperitoneal procedures defined for children. Vessel sealing energy devices (e.g., LigaSure™, Harmonic Scalpel™) were employed to control minor bleeding and prevent thermal injury to adjacent organs. Additionally, all other necessary steps were performed as required for each case.

### Lymph node sampling and tumor retrieval

Lymph node (LN) evaluation and sampling were attempted in all patients who underwent radical nephrectomy. Retroperitoneal lymph node sampling began at the level of the renal hilum. All visible lymph nodes inferior to the renal vein and lateral to the aorta/inferior vena cava (depending on side of tumor) were clipped and removed. Inter-aortocaval dissection was avoided except to remove a suspicious lymph node. In other tumors, the fat and lymphatic tissue along with the tumor specimen was sent for histopathology besides any visible lymph nodes in the surgical field. Tumor spillage was prevented by the use of blunt tipped prograsp & bipolar forceps for maximum control during dissection, careful handling and two experienced surgeons working jointly on console and table. Tumors were safely captured using an ENDOCATCH^TM^ bag inserted through the assistant port and the specimen was retrieved through an infraumbilical low crease (Pfannenstiel) incision ensuring no contact of the tissue with the incision margins.

### Data collection and follow-up

Epidemiological patient data, perioperative data (date, nature of procedure, docking time, total operative time, intraoperative complications, conversion), and postoperative data [complications, length of hospital stay (LOS), pain and analgesic requirement, re-exploration] were collected. Tumor characteristics (nature, size, and histology), adjuvant therapy, and oncologic outcomes were recorded in a database. The Clavien-Dindo (CD) classification was used to categorize postoperative complications, and their management was specifically recorded. Patients had access to the PICU for postoperative care. Follow-up was conducted according to the standard SIOP protocol.

### Primary and secondary objectives

The primary objective was to analyze the role of robotic surgery in managing pediatric solid tumors, in terms of (1) the ease and adequacy of complete or R0 resection, (2) morbidity and mortality related to the procedures, and (3) the long-term oncological outcomes. The secondary objective was to evaluate the benefits and complications of robotic surgery and compare the results with published long-term outcomes of pediatric solid tumors.

### Ethical considerations

This study did not involve a formal institutional ethics committee approval, as it was not a prospective trial. However, all cases were discussed and managed with oversight from our tumor board, which included a medical oncologist, a radiation oncologist, and a robotic surgeon. Written informed consent was obtained from the parents or legal guardians after providing, a detailed explanation of the study procedures, potential risks, and benefits. The study adhered to the principles outlined in the Declaration of Helsinki and followed good clinical practice guidelines.

## Results

### Patient demographics and tumor characteristics

The study included 20 children representing 21 solid abdominal tumors that met the inclusion criteria and underwent surgery between 2013 and 2023. During the same period, 84 pediatric solid tumors were operated by the open surgical approach. The cohort comprised 9 boys (45%) and 11 girls (55%), with a median age of 3.5 years (range: 0.5–13 years). The weight of the children ranged from 7 to 45 kg, with a median weight of 14.6 kg. The smallest patient who underwent successful surgery weighed 7 kg.

### Operative details and postoperative outcomes

All procedures were carried out by a single surgical team at a single institution, using the da Vinci Si Surgical System (Intuitive Surgical, Inc., California, USA). The median operative time was 114 min (range: 30–150 min). The LOS ranged from 2 to 5 days, with a median of 3 days. Conversion to open surgery was required in two cases (10%), due to the aggressive nature of the disease and inadvertent vascular injury. None of the patients required opioid analgesics for pain relief beyond 24 h postoperatively.

### Mortality

Two patients (10%) died during the study period, one with Wilms tumor (WT) and one with malignant pheochromocytoma (PCC). The patient with WT was a 14-month-old boy who presented with dyspnea nearly one year after surgery and was found to have extensive lung metastasis on chest x-ray. One patient with malignant PCC, an 8-year-old boy, died nine months after surgery due to spinal metastasis and respiratory failure.

### Renal tumors

**Figure 2:**
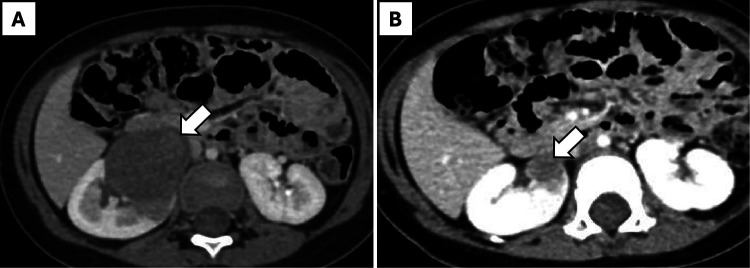
A 12-month-old girl with WAGR syndrome and right-sided Wilms' tumor. **(A)** Pre-chemotherapy axial CT scan showing a large renal mass (arrow). **(B)** Post-chemotherapy axial CT scan demonstrating regression of the mass (arrow).

Nephrectomy was performed after tumor shrinkage to the proposed maximum diameter on contrast-enhanced computed tomography (CECT) scan, achieved within 4 cycles of neoadjuvant chemotherapy in all cases. R0 resection was accomplished in all cases, with tumor sizes ranging from 1.9 cm × 1 cm × 1 cm to 6 cm × 5 cm × 5 cm. Eight children with renal tumor had classical WT, while one girl (aged 7 years, weight 43 kg) had a cystic partially differentiated WT of the upper pole of the left kidney and underwent partial nephrectomy. Robotic surgery was completed successfully in 7 out of 9 patients without complications, with a median operative time of 128 min (range: 102–150 min). Estimated blood loss ranged from 10 to 105 ml, with a median of 20 ml. Conversion to open surgery was required in the patient with cystic partially differentiated WT due to intraoperative injury to the upper polar vessel. Another patient experienced intra-abdominal renal capsule rupture with a small and localized tumor-spill (Table 1). This was associated with limited spillage of necrotic content. One girl (aged 12 months, weight 12.6 kg) with WAGR syndrome and right-sided WT (Figure 2) underwent nephron-sparing surgery (NSS) but experienced a urine leak through the drain (CD grade 1), which resolved with conservative management after 2 weeks.

**Table 1:** Epidemiological data and results of Wilms Tumor patients who underwent robotic nephrectomy and lymph node sampling.

Sr.	Age (months)	Sex	Weight (kg)	Side	Surgico-pathological staging	Procedure	Total operative time (min)	Estimated blood loss (ml/kg)	Intra-operative and postoperative complications	Conversion to open surgery	Tumor size (cm)	Gross margins	Follow up (years)	Disease free at last follow up
1	6	M	7	Left	Stage 3	Radical Nephrectomy + LN Biopsy	135	2.86	Capsule Rupture	No	2.5 × 2 × 2	Free	7	Yes
2	9	F	8	Right	Stage 1	Radical Nephrectomy + LN Biopsy	145	1.25	Postoperative Fever (CDC grade 1)	No	5 × 1 × 5	Free	4	Yes
3	12	F	12.6	Right	Stage 1	Right Partial Nephrectomy	130	2.78	Urine Leak (CDC grade 2b)	No	2 × 2 × 1	Free	6.6	Yes
4	12	F	7.8	Right	Stage 1	Radical Nephrectomy + LN Biopsy	120	1.92	No	No	2 × 2.5 × 1	Free	4.4	Yes
5	14	M	12	Left	Stage 1	Radical Nephrectomy + LN Biopsy	110	4.17	No	No	6 × 5 × 5	Free	1	No^a^
6	24	M	13.2	Right	Stage 1	Radical Nephrectomy + LN Biopsy	102	1.14	No	No	5 × 5 × 5	Free	6	Yes
7	36	F	13.6	Right	Stage 1	Radical Nephrectomy + LN Biopsy	110	1.47	No	No	4.5 × 5 × 4	Free	1	Yes
8	36	M	12.6	Left	Stage 1	Radical Nephrectomy + LN Biopsy	118	1.59	No	No	4 × 5 × 3	Free	2	Yes
9	48	F	16.2	Right	Stage 1	Radical Nephrectomy + LN Biopsy	118	1.23	No	No	4.7 × 1.5 × 1.5	Free	3	Yes
10	84	F	43	Left	Stage 1	Right Partial Nephrectomy	150	2.44	Injury To Upper Polar Vessel	Yes	1.9 × 1 × 1	Free	3	Yes

M, Male; F, Female; LN, Lymph Node; CDC, Clavien-Dindo Classification.

^a^ Died of extensive lung metastases 1 year after surgery.

Surgico-pathological staging revealed that all tumors were Stage 1, intermediate-risk group, except for the partially differentiated cystic variety, which was low-risk and the one with tumor rupture (Stage 3). All surgical margins were free of tumor. There was one case for partial nephrectomy for Syndromic Wilms tumor, where frozen section was of special relevance, and no difference was found on the result of frozen section vs. and histopathology of specimen. The number of lymph nodes (LN) sampled ranged from 2 to 7, with a median of six nodes.

One girl required intravenous antibiotics for postoperative fever, while the remaining eight received oral antibiotics for five days postoperatively. The median LOS was 3 days (range: 2–5 days). No tumor recurrence was noted for the patient with intraoperative tumor spill.

### Adrenal tumors

**Figure 3:**
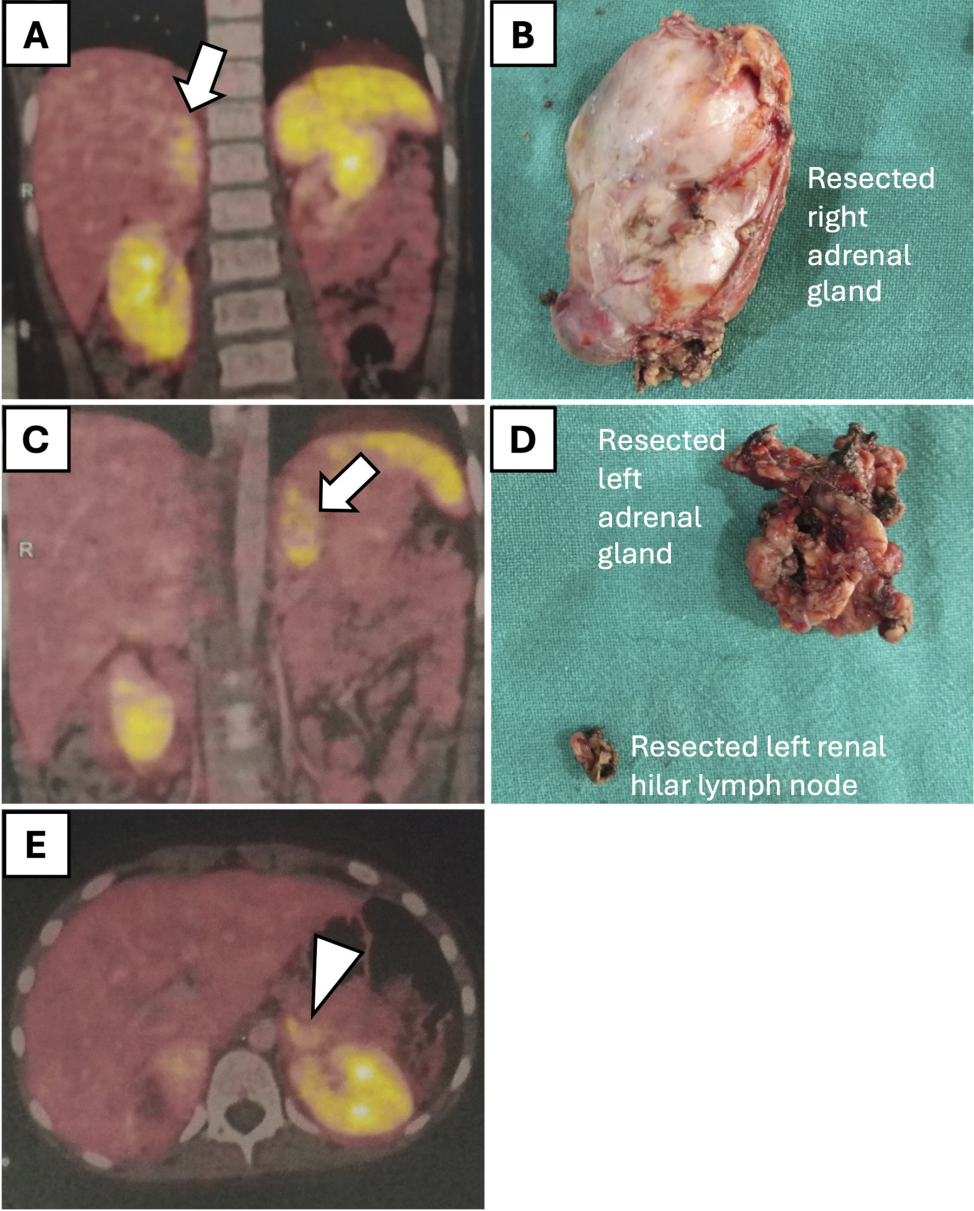
Preoperative 68Ga-labeled somatostatin analogue (DOTA-peptide) positron emission tomography-computed tomography (PET-CT) scan and corresponding histological specimens in a 10-year-old male patient with bilateral pheochromocytoma. **(A)** PET-CT scan showing increased uptake in the right adrenal gland (arrow). **(B)** Resected right adrenal gland specimen. **(C)** PET-CT scan indicating the additional left adrenal lesion (arrow). **(D)** Resected left adrenal gland specimen along with a lymph node near the renal hilum. **(E)** PET-CT scan showing increase uptake in the lymph node near the left renal hilum (arrowhead).

Seven children (4 males and 3 females), aged between 6 months and 13 years, with a weight range of 9.9–45 kg, had adrenal masses (Table 2). One male patient (aged 10 years, weight 36 kg) had bilateral pheochromocytoma (PCC) associated with Von Hippel-Lindau (VHL) disease and underwent bilateral adrenalectomy (right: 135 min, left: 100 min) with an estimated blood loss of 100 ml and intraoperative oozing from the tumor bed which could be controlled. In this patient, a 68Ga-labeled somatostatin analogue (DOTA-peptide) positron emission tomography-computed tomography (PET-CT) scan done preoperatively diagnosed an additional left adrenal lesion that was initially not suspected (Figure 3).

**Table 2:** Epidemiological data and results of patients who underwent robotic adrenalectomy.

Sr.	Age (Years)	Weight (Kg)	Tumor Diagnosis	Procedure	Total Operative time (min)	Estimated blood loss (ml/kg)	Intra-operative and postoperative complications	Conversion	Tumor Size (cm)	Margins	Follow up (months)	Disease free event
1	0.5	9.9	CA	Right Adrenalectomy	111	1.01	No	No	2.2 × 2.1 × 1.5	Free	12	Yes
2	1	11.8	CA	Left Adrenalectomy	100	1.69	No	No	4.5 × 4 × 1.5	Free	60	Yes
3	5	15.6	Neuroblastoma	Right Adrenalectomy	105	0.64	No	No	5 × 4 × 2.6	Free	18	Yes
4	8	22.5	PCC (Malignant)	Right Adrenalectomy	120	1.33	No	Yes	5 × 5.2 × 4	Free	9	Mortality
5	10	36	Bilateral PCC	Bilateral Adrenalectomy	R (135) L (100)	2.78	Oozing from tumor bed (controlled)	No	R-(5 × 5 × 4) L-(2 × 1)	Free	20	Yes
6	10	45	Ganglioneuroma (mature sub type)	Left Adrenalectomy	103	0.22	No	No	4 × 3 × 4.4	Free	24	Yes
7	13	41	PCC	Right Adrenalectomy	124	0.61	No	No	3 × 5 × 4.3	Free	6	Yes

CA, Cortical Adenoma; PCC, Pheochromocytoma; R, Right; L, Left.

The most common adrenal pathology was PCC (*n* = 3), followed by functional cortical adenoma (*n* = 2), ganglioneuroma (*n* = 1), and neuroblastoma (*n* = 1). Five patients had benign adrenal pathology, while two had malignant pathology (neuroblastoma and malignant PCC).

The patient with malignant PCC (aged 8 years, weight 22.5 kg) required conversion to open surgery due to extensive involvement of surrounding vascular and vital structures, which hindered progression via the robotic approach. The intense desmoplastic tissue reaction was not appreciable on the preoperative CT scan. Although the tumor was resected completely and the margins were negative on pathology, this patient died nine months later due to spinal metastasis and respiratory failure, unrelated to the mode of surgery the patient underwent. Apart from this conversion, the remaining robotic adrenalectomies were completed without complications, with a median operating time of 108 min (range: 100–124 min). The median operative blood loss was 20 ml (range: 10–30 ml), and the median LOS was 2 days (range: 1–3 days). Tumor sizes ranged from 2.2 cm × 2.1 cm × 1.5 cm to 5 cm × 5.2 cm × 4 cm, with free surgical margins in all cases.

### Retroperitoneal masses

**Figure 4:**
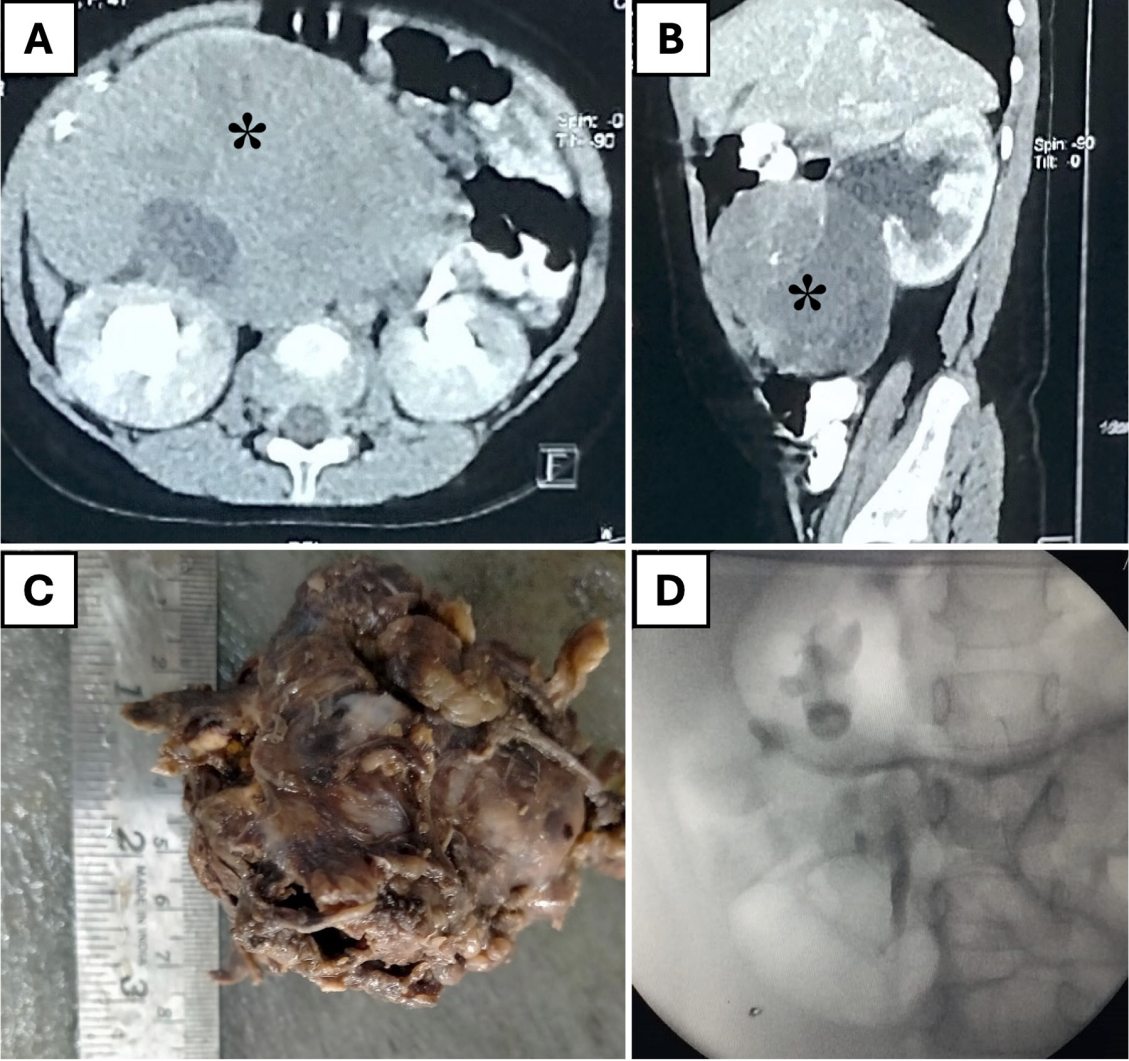
A 4-year-old girl with an extrarenal Wilms' tumor. **(A)** Axial CT scan showing a large, heterogeneous retroperitoneal mass (*asterisk*). **(B)** Sagittal CT scan revealing the mass (*asterisk*) separate from the right kidney and causing hydronephrosis. **(C)** Resected tumor specimen. **(D)** Postoperative retrograde pyelogram demonstrating an intact pelvicalyceal system on the right side.

Among the three patients with retroperitoneal masses, two patients with opsoclonus-myoclonus syndrome were diagnosed with paraspinal neuroblastoma (NBT), and one girl had an extra-renal WT on the right side. The 4-year-old girl with extrarenal-WT presented with chronic constipation for one year without fever, weight loss, or bladder issues. Imaging revealed a large, heterogeneous retroperitoneal mass causing bilateral hydronephrosis. A biopsy identified an extrarenal Wilms tumor. She underwent six cycles of neoadjuvant chemotherapy, followed by robotic-assisted tumor excision and stenting of the right ureter. Operative findings included tumor infiltration and adherence to the right ureter but the major vessels were preserved. Post-surgery, she underwent radiotherapy. Follow-up retrograde pyelogram revealed preserved right ureter and kidney (Figure 4). Following a comprehensive radiological and metastatic workup, the remaining two patients underwent upfront surgery. The masses were successfully resected *en bloc* without complications. The average operative time for removing these retroperitoneal masses was 93 min, with an average estimated blood loss of 25 ml.

## Discussion

Solid tumors account for 60% of pediatric cancers (11), with neuroblastomas (NBTs), and Wilms tumors (WTs) being the most common intra-abdominal solid tumors. The five-year survival rate for children with solid tumors has significantly improved to 75%–80% (12), owing to advancements in multimodal treatment approaches, including surgical resection as the primary treatment modality, traditionally performed via open surgery (13). This increase in survival rates has prompted efforts to improve survivors' quality of life by reducing treatment-related morbidity through the adoption of minimally invasive surgical (MIS) techniques, while continuing to enhance outcomes in selected relatively low-risk patients.

Robotic surgery has been widely adopted in the adult population; however, its application in pediatric urologic oncology remains limited due to several factors, including the rarity of pediatric genitourinary malignancies, the higher risk associated with tumor surgery in the pediatric population, and concerns over adhering to oncosurgical principles with MIS (2, 3, 14). Recent case reports and case series have demonstrated the safe and practical use of robotic-assisted surgery in excising pediatric solid tumors, following the initial report by Gutt et al. (15). Our case series presents the excision of pediatric abdominal solid tumors using robotic surgery, with favorable perioperative outcomes in terms of blood loss, complications, and conversion rates, as well as encouraging oncological outcomes regarding surgical margins, nodal counts, and disease-free intervals.

### Renal tumors

The size and extent of the tumor are crucial factors to consider when performing MIS due to concerns of tumor spillage. Dissecting tumors in small intraperitoneal spaces while avoiding collateral damage becomes increasingly challenging, especially in cases where large tumor masses cross the midline, posing an increased risk of tumor rupture, which can lead to upstaging and disease progression (16). However, there are conflicting reports on the maximum size of tumors that can be safely removed using MIS. According to Duarte et al., the largest diameter tumor that can be safely resected via the MIS approach was equivalent to 10% of the patient's height, and they were able to excise tumors as large as 12 cm in diameter (17). In contrast, a multicenter trial by Varlet et al. concluded that tumors with a maximum diameter of 8 cm and not crossing the midline could be safely removed (18). In our initial experience, satisfactory outcomes were achieved for tumors less than 6 cm in maximum diameter, with the added benefit of preoperative chemotherapy.

One case in our series reported a breach of the tumor capsule, which led to a localized spillage of necrotic tumor content. This was among the first five cases of the series. With time, we were able to reproduce results similar to those of open surgeries. The median operative time for nephrectomy was 128 min, shorter than reported in other studies, which we attributed to the selection of smaller tumor diameters and our increasing experience with robotic tumor surgery (4, 19).

Accurate staging requires sufficient lymph node (LN) sampling to avoid tumor under staging and the risk of local recurrence (20). While it is commonly believed that MIS does not allow for adequate LN sampling (17), our experience shows that the robotic approach enables LN harvesting similar to recent MIS and open surgery series, with a median of 6 LNs sampled (17, 19, 21, 22).

Nephron-sparing surgery (NSS) was attempted in two patients: one with WAGR (Wilms Tumor, Aniridia, Genitourinary anomalies and intellectual disability) syndrome and right unilateral WT, and the other with a cystic nephroma. NSS for unilateral WT has long been accepted in syndromic patients and has recently been extended to any WT that meets the Umbrella protocol criteria (23). Open surgery is currently the standard of care for NSS in children with WT (24). Robotic-assisted NSS for WT has been reported infrequently (6, 14). Our first experience with robotic NSS after neoadjuvant chemotherapy was promising, and it appeared desirable in syndromic WT cases. The robotic approach provided the dexterity required for vascular isolation, parenchymal division, collecting system repair, and free suturing of the renal bed without the need for vascular clamping. While performing NSS on a patient with a cystic nephroma, bleeding from the upper polar vessel occurred, leading to conversion to open surgery. An upper pole nephrectomy was attempted in this case owing to the small tumor size (4 cm) and non-hilar location. In the literature, conversion rates of up to 29% have been reported for radical nephrectomy (19). There is insufficient data for meaningful comparison of conversion rates in partial nephrectomy via robotic approach in children.

At the end of 10 years, with a median follow-up of 5 years, the overall survival (OS) [90%] and event-free survival (EFS) [90%] were comparable to the outcomes of stage 1 favorable histology tumors excised via an open route in low and high-income countries (24, 25). We had modest blood loss and a LOS equivalent to adult literature in this series. Robotic nephrectomy allowed the accomplishment of complex tasks in a minimally invasive manner, following the same staging and oncological dissection rules as open surgery, with the benefits of shorter LOS and earlier initiation of adjuvant therapy. Our results are encouraging and comparable with previously published series of MIS for nephrectomy in WT (Table 3).

**Table 3:** Our robotic radical nephrectomy with LN sampling and adrenalectomy experience compared with published literature on minimally invasive radical nephrectomy and adrenalectomy.

Studies on radical nephrectomy	Sample size/approach	Median operative time (min)	Complications (*n*)	Conversion rate (%)	Lymph nodes sampled	Median hospital stay (days)	Follow up (months)
Blanc et al. (19)	10/Robotic	270	None	30	6	4	16 (1 death)
Duarte et al. (12)	24/Lap	165 ± 27	Incisional hernia (1)	–	2.52 ± 2.08	2.3	78 (2 relapses)
Andreas et al. (25)	9/Lap	147	None	–	2	–	48
Varlet et al. (18)	16/Lap	124	Small bowel perforation (1), Tumor rupture (1)	6	3.4	3	42 (1 local recurrence, 1 death from distant brain metastasis)
Bouty et al. (20)	50/ (46Lap + 4Robotic)	194	Bowel, spleen and renal vein injury (1 each)	12	4	4	34 (2 local recurrences and 1 metastatic relapse)
**Current series**	**9/Robotic**	**128**	**Vessel injury (1), Urine leak (1)**	**11**	**6**	**3**	**58 (1 death)**
Studies on adrenalectomy	Sample size/approach	Median operative time (min)	Complications (*n*)	Conversion rate (%)	Lymph nodes sampled	Follow up (months)	
Blanc et al. (26)	24/Robotic	–	Retroperitoneal collection (1)	4	4	–	
Mitra et al. (27)	3/Robotic	244	None	–	–	19	
Kadamba et al. (28)	10/Lap	141	None	–	–	24 (1 death)	
Girolamo et al. (29)	68/Lap	170 ± 87	Intraoperative bleeding requiring transfusion (5), Tumor rupture (1), Diaphragmatic tear (1)	–	4.2 ± 2.5	52 (2 recurrences)	
**Current series**	**7/Robotic**	**108**	**None**	**1/7(14)**	**3**	**22 (1 death)**	

The bold values represent the data from our (authors') series.

### Adrenal tumors

Since the first report of transabdominal laparoscopic adrenalectomy by Gagner et al. in 1992 (31), MIS has become the standard approach for adrenalectomy. Robotic adrenalectomy was first introduced by Piazza et al. in 1999 and has been widely adopted by urologists due to its low complication rate (32). However, the literature on robotic adrenalectomy for the pediatric population is limited, with few case reports and series reported to date (28).

We demonstrate that robotic adrenalectomy is feasible in children of different ages and body types. The median operative time for robotic adrenalectomy in our series was 108 min, similar to the adult equivalent reported by Brunaud et al. (33) and shorter than prior pediatric adrenalectomy studies (4, 28–30, 34). We had no unexpected intra- or postoperative complications, possibly due to the stereoscopic magnified vision that facilitated the identification and dissection of small adrenal veins.

In our series, one case of pheochromocytoma (PCC) was converted to open surgery due to its infiltrative character, later confirmed as malignant PCC. None of our PCC cases experienced intraoperative hypertensive crises. Our experience with synchronous bilateral transperitoneal total robotic adrenalectomy in a patient with PCC and Von Hippel-Lindau (VHL) disease was pleasing, with a total operative time of 235 min. The choice to proceed with bilateral total adrenalectomy rather than cortical-sparing adrenalectomy was made due to elevated urine dopamine levels and tumor size, both of which significantly enhance the risk of malignancy (35).

The median LOS for our robotic adrenalectomy series was 3 days, comparable to prior reports (Table 3). Our series represents the longest reported follow-up of pediatric patients undergoing robotic adrenalectomy. With the transperitoneal approach, we achieved excellent exposure and adequate working space, demonstrating its oncological safety and efficacy in pediatric patients. Although a retroperitoneal approach to adrenalectomies has been described, we chose to focus on expanding our experience with the transperitoneal approach.

### Neuroblastomas

Opsoclonus-myoclonus syndrome is often linked with L1 paraspinal neuroblastomas (NBTs), which can be effectively treated with simple robotic resection. In our series, two girls with this syndrome underwent robotic excision without complications and with favorable long-term outcomes. Case selection for robotic excision is crucial given the infiltrative nature and advanced stage at presentation of NBTs. The L1 stage disease, with a small tumor diameter, allowed for safe dissection without adjacent organ injury. However, Brisse et al. have defined the role of MIS in IDRF positive patients as well (34). According to studies conducted by Nabel et al. and Thomas et al., robotic surgery seems to be as effective as open surgery in appropriately selected cases of NBTs (7, 27).

With the rapid evolution of robotics in pediatric surgery and the encouraging results of various studies, including ours, robotic surgery could be a useful tool in properly selected pediatric oncology cases. However, more research is needed to evaluate its robust utility and safe practices in pediatric oncology. We had modest blood loss and a LOS equivalent to adult literature in this series. The lack of tactile feedback was partially compensated by enhanced optics. While our data was limited to a single center, similar results have been reported in other published series from multiple centers (Table 4) (18, 26, 29, 36). Nonetheless, the application of robotic surgery in pediatric oncology is not without challenges. The learning curve of robotic surgery for pediatric tumors can be longer as compared to the benign indications as the margin of error is lower. Concerns regarding the potential for tumor spillage or seeding during minimally invasive procedures remain a valid consideration, particularly for larger or more advanced tumors (2, 5, 16). Additionally, the rarity of pediatric genitourinary malignancies and the associated learning curve may limit the widespread adoption of robotic techniques in this field (14).

**Table 4:** Comparison of our study to the other with the other published data on robotic surgery in pediatric solid tumors.

Study	No of Procedures	Median age (years)	Conversion (%)	Median Follow up (years)	Year
Meehan et al. (6) (unicentre)	14	NA	29	NA	2008
Meignan et al. (4) (bicentre)	11	7.6	8	3.5	2018
Varda et al. (37) (unicentre)	8	14	0	NA	2018
Navarrete et al. (38) (unicentre)	5	NA	NA	2	2019
Blanc et al. (27) (multicentre)	93	8.2	8	2.4	2022
Our series (unicentre)	**20**	**2.5**	**10**	**2**	**2024**

The bold values represent the data from our (authors') series.

It is crucial to carefully select suitable cases for robotic surgery, considering factors such as tumor size, location, extent of disease, and the surgeon's experience. Appropriate case selection, adherence to oncological principles, and meticulous surgical technique are essential to ensure favorable outcomes and minimize complications (18, 34). Despite the limitations of our single-center study, our results contribute to the growing body of evidence supporting the feasibility and safety of robotic surgery in the management of selected pediatric solid tumors. Larger, multicenter studies with longer follow-up periods are warranted to further validate the oncological outcomes and establish best practices for the integration of robotic techniques in pediatric oncological surgery.

## Conclusion

In conclusion, this study demonstrates the feasibility and safety of robotic surgery for the management of select pediatric solid abdominal tumors. Our experience over a decade showcases favorable perioperative outcomes, including modest blood loss, low complication rates, and a length of hospital stay comparable to adult literature. Oncological principles of complete resection and adequate lymph node sampling were upheld, resulting in encouraging long-term disease-free survival rates on par with open surgery. While larger multicenter studies are warranted, robotic surgery emerges as a promising minimally invasive approach in pediatric oncology, potentially offering improved cosmesis and earlier recovery without compromising oncological outcomes in appropriately selected cases. Careful patient selection and adherence to surgical principles remain paramount for successful integration of this innovative technology.

## Data Availability

The raw data supporting the conclusions of this article will be made available by the authors, without undue reservation.
